# The Application of Acceptance Models to Human Resource Information Systems: A Literature Review

**DOI:** 10.3389/fpsyg.2021.659421

**Published:** 2021-05-31

**Authors:** Lou Menant, Daniel Gilibert, Céline Sauvezon

**Affiliations:** Laboratoire Epsylon EA 4556, Université Paul Valéry Montpellier III, Montpellier, France

**Keywords:** acceptability, user acceptance, human resource information system, unified theory of acceptance and use of technology, technology acceptance model

## Abstract

Technology acceptance by users has been extensively studied in recent years in various fields such as technologies for learning, e-commerce, and business technologies. This review focuses specifically on Human Resource Information Systems (HRIS) and its acceptance by users. Given their widespread use in organisations, HRIS acceptance has been researched but not synthesised in any way. This article aims to review the effectiveness of the classical TAM and UTAUT models commonly used for new technologies and to identify the variables added to these models to better predict HRIS acceptance by employees. It also highlights the importance of the human-machine-organisation relationship to contribute to the understanding of HRIS acceptance in professional environments. This review confirms the effectiveness of the TAM and UTAUT models and proposes to develop them by (a) variables reffering to technological characteristics (security, system response time, and the data quality implemented in the system), (b) user satisfaction with the system, and (c) organisational variables (expected role of the HR department). The discussion focuses on the retroaction possibilities between the different Human-Machine-Organisation relation levels.

## Introduction

Technologies are omnipresent in all professional spheres, including the field of human resource management. These technologies can be found under different names in the HRIS literature. (e-HR, by Panayotopoulou et al., 2007 and Ramirez, 2002; e-HRM, by Huang and Martin-Taylor, 2013 and Saleh and Saleh, 2016; Human Resource Information Systems, by Mahadik and Ayarekar, 2020, and Chen and Gaffney, 2020). These have been the subject of numerous studies in the fields of the sciences of management, computers, and psychology (Stone et al., 2006, 2015; Al-Dmour et al., 2013; Chakraborty and Mansor, 2013; Bondarouk et al., 2017; Qadir and Agrawal, 2017). These systems allow all or part of Human Resource Department activities to be made paperless via computerised systems, sometimes interconnected. They offer solutions for various activities: the management of jobs and skills in the organisation, regular interviews and recruitment interviews, professional mobility, the monitoring of working hours and activities, management of paid leave, refunds of expenses, etc.

### Human Resource Information Systems

Human resource information systems have been defined by several authors. Tannenbaum (1990) defines them as a system used for acquiring, storing, handling, analysing, sorting, and distributing relevant information related to human resources in an organisation. In (Hendrickson, 2003) extended this definition of HRIS by describing them as systems which consist of processes, procedures, persons and functions for the acquisition, conservation, recovery, analysis, handling, and distribution of information relative to an organisation's human resources. More recently, Ruël et al. (2011) proposed a definition introducing the concept of information systems or computer applications, playing a facilitating role in the practise, policy, and strategies of human resource management. Voermans and van Veldhoven (2007) propose a simple definition. In fact, according to them human resource information systems are administrative and technological aids for carrying out HR tasks. The term HIRS therefore encompasses a large number of applications such as “*recruitment management, induction, pay, company incentives, professional appraisal, training, career management, mobility, skills and talent management, succession planning, and company charts…”*(Geuse, 2007). So a Human Resource Information System (HRIS) can be defined as a facilitating computerised system which supports information management and administrative and strategic tasks as well as decision-making by human resource departments.

Thus, the purposes of an HRIS and the issues arising therefrom encourage a specific interest in this type of technology. Indeed, a major reason for focusing on HRIS is that these applications speed up and have a considerable influence on decisions which can be taken in relation to employees. By facilitating access to data, they influence the information which is taken into account. The use of an HRIS, by speeding up the processing of administrative information can allow Human Resource Managers to concentrate their efforts on other HR roles (Ulrich, 1997) such as that of support for change within the organisation, that of a strategic partner and monitoring the well-being of employees (Hussain et al., 2007). Thus, HRIS alter the role of human resource managers and departments in their relations with employees and their way of carrying out their duties (Kolatshi, 2017). HRIS are also specific in that these technologies are not based on spontaneous use by the employee, as this does not depend solely on their desire to do so (Yoo et al., 2012; Sun et al., 2014). They also directly affect many determinants of employees' career development, as well as their conditions of employment. Finally, HRIS change the modalities of social interaction within the organisation, between employees and in the relationships between employees and Human Resource Managers. In this context, understanding employees' acceptance or non-acceptance of HRIS is a complex issue, involving individual and technological factors, intertwined in an organisational and social context (Laval and Guilloux, 2010; Bondarouk et al., 2017).

### Technology Acceptance

Many studies have questioned the factors which influence the adoption and acceptance of HRIS. According to Strohmeier and Rudiger (2009), research on this subject is generally divided into two fields of study: the adoption of these technologies on behalf of organisations by decision makers and their acceptance by employees. Their adoption by organisations has already been the subject of research syntheses (Al-Dmour et al., 2013; Stone et al., 2015) which is not the case for acceptance by employees and for their satisfaction, which is however decisive for the use of these technologies by employees, over time in the context of their work.

To define what acceptance is, it would be useful to recall the words of Bobillier-Chaumon and Dubois (2009). In fact, these authors tackle the concept of acceptance via two interdependent approaches. One “*concerns the system and its characteristics*” (Dubois and Bobillier-Chaumon, 2009; p. 306) which must be consistent with the expectations and requirements of the user. This approach mainly highlights the notions of the technology's usefulness and usability. A useful system meets the requirements and expectations of the users in terms of functionalities and objectives being pursued. Usability refers to the ability of a system to respond via its technical and ergonomic properties to the individual characteristics of its users to perform a given task in a specific context. Thus, this approach focuses on the adequacy of the functional, technical, and ergonomic aspects of the technology to the specificities of its context of use. This first approach is so complemented by a “*user-focused approach*” to the technology and more specifically one focused on the way in which they perceive it, understand it and choose to use it (cognitive ergonomics, a priori acceptance, and actual acceptance). It relates therefore to the agency of the employee faced with Information Systems. The user-centred approach takes into account the individual aspects to which the technological features should ideally be adjusted. Thus, optimal acceptance can be defined as the result of these two elements: a technology with characteristics which are compatible with the objectives of the user and, with regard to the user, psychosocial tendencies which are favourable to the implementation of new behaviours, so that the technology can be adopted and used. In this context, acceptance appears to require a combination of factors linked to the characteristics of the technology, those of its user and implicitly to the organisational context of use (social influences and facilitating conditions within the organisation).

### Interaction Between Human, Technology, and Organisation

So the authors asked themselves the crucial question of what causes the development of an information technology solution to succeed or fail within work organisations (Human-Organisation-Technology-Fit, Erlirianto et al., 2015; Human-Technology-Organisation Symbiosis Model, Brangier, 2003). Historically, while the question of the intention to use was a prerequisite for the development of computer programming, today it is considered that actual use is dependent on the organisational efficacy of the solution (enabling the achievement of the operational objectives pursued within the organisation, Panos and Bellou, 2016). These information technology solutions have now become a permanent feature in the everyday lives of employees, as they are omnipresent and above all indispensable for the performance of individual and collective activities (standardisation of work procedures, remote interactions). Information technology tools, which simply made it possible to exceed the limited calculation capacities of human beings, have moved from the role of assistant to that of manager of their activities. They track activities, request them, and manage them through algorithms (automatic reminder e-mails, platform tracking progress in carrying out an activity, automatic skills analysis based on an algorithm). As interaction with the technology is unavoidable, the issue at stake becomes that of the employee's satisfaction/dissatisfaction, which could provoke a desire to leave the organisation (Kolatshi, 2017) and/or feedback on the information system with the aim of developing its functionalities, usability, and its fit with the operations and needs of the organisation. On this subject, Brangier (2003) mentions different types of feed-backs: those aiming to regulate the adaptability of the information system to the situation via new functionalities, those aiming to regulate human psychological and psychosocial processes when confronted with technology; and finally collective feed-backs via organisational changes in response to the introduction of the new system. These feed-backs in fact correspond to the historical evolution of studies on Information Systems (Clegg, 1994): the first concerned the ergonomics of design and use and the last social and managerial sciences. New HR technologies are already present and unavoidable in organisations. As a result, it is conceivable that intentions at work only partially influence actual use, since it is required to do the job. Satisfaction with use could be closely related to the actual use of work technology, and a central variable at the individual level, requiring feed-back loops to be maintained. The feedback loops between the three interdependent levels of analysis: technology, human and organisation, in the unique context of HRIS, invite a review of the acceptance of this technology, as an element of the relationship between the employee and the organisation, and of the expectations concerning the roles of human resources management, which are, of course, administrative, but also involve support for change, strategy, and monitoring of the quality of life of employees. This is within this global conceptual framework that this critical synthesis of current studies on the acceptance of HRIS takes place and makes proposals on the factors that could to be considered in future studies.

### Aims and Contribution

In accordance with the recommendations of Venkatesh et al. (2016) and Workman (2014), who establish the need and value of contextualising the acceptance of a technology according to its specific objectives, this review focuses on the acceptance of human resource information systems. In this way, it allows the specificities of HRIS compared to other technologies to be taken into account. This narrative literature review could provide researchers and protagonists interested in these issues with a synthesis incorporating earlier research. To this end, we are pursuing a 3-fold objective: to review the relevance of the classical TAM and UTAUT models for predicting employee acceptance of HRIS, to propose a review of specific variables to SIRH which until now have been studied in a disparate way in isolated studies and finally to better highlight the necessary articulation between Humans, machines and organisational context in understanding the HRIS acceptance process.

In order to meet this objective, we will list the factors influencing the acceptance of HRIS by structuring our presentation in two stages. We first looked at what was proposed by the previous literature concerning the application of UTAUT to HRIS, hence a focus on articles dealing with utility and ease of use. We also included in our review articles based on the TAM model, predecessor of the UTAUT model, and which provided answers to the recommendations of Venkatesh et al. (expected role of the HR department, impact of the national context, social influence). Twenty-three studies were taken into account, the first presented above were based on the traditional TAM model and the following based on the integrative UTAUT model. These analyses will allow us to closely examine the relevance of the central variables of these models in the specific context of Human Resource Information Systems. It will also involve highlighting the additions suggested by authors in order to identify the issue of the acceptance of HRIS. We will conclude by defending the interest of considering interdependence between contextual factors, individual factors, and technological characteristics.

## Summary of the Main Results From the Literature

Among the technology acceptance models, the TAM (Davis, 1989) is one of the original models and the UTAUT model (Venkatesh et al., 2003) is the one which most incorporates other models. They are particularly well-represented in the literature (Benbasat and Barki, 2007; Brangier and Hammes-Adelé, 2011), for their ability to predict the final stage of acceptance of a technology, namely its use (Amiel and Van De Leemput, 2014)^1^. This high level of representation and the consensus concerning their ability to explain a part of the variance in relation to the acceptance of technology in general and more specifically of HRIS, led us to choose these models when constructing our presentation. These two models feature a common approach to the acceptance of technologies. In fact, the variable of perceived ease of use and that of effort expectancy share an identical definition in both models. The same is true for the variable of perceived usefulness and that of performance expectancy. These models both also originate in the theory of reasoned action, explaining the actual use of technology by the effect of individual explanatory variables on intention to use. These similarities can be explained by the method used to construct the UTAUT. In 2003, Venkatesh et al. observed that there were a multitude of models in existence to explain the acceptance of technologies. They therefore suggested constructing a unified theory based on eight models including the TAM. The UTAUT can therefore be considered as a theory which incorporates the TAM but which expands it with the variables of “facilitating conditions” and “social influence.” Although the TAM has been subject to many criticisms in the literature such as the inconsistency of the link between intention to use and actual use (Bagozzi, 2007; Nistor et al., 2013; Harrison et al., 2014; Nistor, 2014), the lack of formalisation of antecedents to beliefs (Benbasat and Barki, 2007) and even the reduction of acceptance to frequency of use (Benbasat and Barki, 2007; Schwarz and Chin, 2007), the Davis model is today still the subject of research in the field of the acceptance of HRIS (Bayraktaroglu et al., 2019; Shahreki et al., 2020). We have therefore chosen to retain the presentation of this model. The results presented in Tables 1, 2 support the relevance of the common variables. They also confirm the interest of variables relative to technology and user satisfaction which has already been observed in other contexts (Venkatesh et al., 2016). Finally, they support the proposal to consider organisational context variables in the study of technology acceptance from (Venkatesh et al., 2016).

**Table 1:** Synthesis of studies based on the TAM model (Davis, 1989) explaining the acceptance of an HRIS technology for users.

**References**	**Sample**	**Country**	**Proposed extension of the TAM model**	**Main results**
Voermans and van Veldhoven (2007)	356 managers and employees	The Netherlands	Ulrich's model of Human Resource roles (Ulrich, 1997) Conviviality of the system Quality of assistance to users Quality of the system	The preference for an HR role as a strategic partner predicts a **positive attitude to the tool**. The preference for an HR role of support for employees, and guarantor of quality-of-life, is linked to **a negative attitude to the tool**. The perceived usefulness, conviviality, quality of assistance, and quality of the system predict a **positive attitude to the tool**.
Huang and Martin-Taylor (2013)	258 employees from a construction company	England	Quality of the data contained in the system Earlier favourable or unfavourable experiences Training Involvement of users	Ease of use, perceived usefulness, data quality, in-depth training, and results obtained in the past via the system predict the **use of the system**. NB: diachronic study in the form of qualitative and quantitative action research
Abdulah et al. (2013)	40 SME managers	Malaysia	Gender Age Education	Gender, age and education are not significantly linked to **perceived usefulness** and **perceived ease of use**.
Bamel et al. (2014)	90 university professors	India	Determinants of user satisfaction according to Haines and Petit (1997)	The main **perceived advantages of the use of an HRIS** are speed of response, access to information, the improvement of services to employees and the reduction of administrative paperwork. **The main perceived obstacles to the use of an HRIS** are a lack of support from management, a poor perception of use, and finally a lack of computer knowledge and expertise.
Amiel and Van De Leemput (2014)	999 managers and employees	Belgium, France, Italy and the USA	National context Command of the language used in the system	The national context leads to differences in the perception of **ease of use, usefulness, conviviality, performance of the tool, and to different frequencies of use**. The level of command of the English language used in the system affects **perceived usefulness and ease of use**.
Panos and Bellou (2016)	80 HR managers	Greece	Impact of objectives linked to Human Resource roles based on Ulrich's model of Human Resource roles (Ulrich, 1997).	**Perceived usefulness, perceived ease of use and attitude to the system** have a positive impact on the results obtained by the HRIS. **Perceived usefulness, perceived ease of use and attitude to the system** are positively correlated with the fact that HR managers are pursuing relational and transformational goals when using the HRIS. The impact of the role attributed to the HR department on the results achieved by the HRIS is moderated by perceived usefulness, perceived ease of use and attitude to the system.
Saleh and Saleh (2016)	490 employees from a service company	Palestine	Yale model of communication and persuasion (Hovland and Janis, 1959)	Perceived security, response time, perceived risk, support from the company and perceived ease of use are predictors of **perceived usefulness**. System response time and perceived risk are predictors of **perceived ease of use**.
				Perceived usefulness, perceived ease-of-use and perceived risk predict a **positive attitude to the tool**. Perceived usefulness, social risk, social influence, support from the company, communication, and attitude predict **intention to use**.
Kolatshi (2017)	258 employees from HR departments without distinction as to position of seniority in the department.	Libya	Information systems success model (Delone and McLean, 1992)	Perceived usefulness, support from management, use of HRIS for strategic activities and social influence predict **satisfaction with the tool used**. The predictive power of perceived ease of use, flexibility of the system and the quality of information on **user satisfaction with the tool** are mediated by perceived usefulness. Perceived usefulness is predicted by support from management, perceived ease of use, flexibility of the system, and quality of information. Satisfaction with the tool predicts **affective involvement and intention to leave the company on the part of Human Resource Managers**.
Kamaludin and Kamaludin (2017)	267 employees of a private hospital	Malaysia	Quality of information User satisfaction Social influence	Perceived ease of use, social influences, and quality of information encourage **use of the technology**. The quality of the information used in the system favours **perceived usefulness**. Use is positively **linked to satisfaction**. Paradoxically, **use of the technology** is negatively correlated with its perceived usefulness^1^.
Bayraktaroglu et al. (2019)	112 employees of an SME	Turkey	Information systems success model (Delone and McLean, 1992)	Satisfaction with the system encourages **use of the HRIS**. Behavioural control, ease-of-use, quality of data, quality of the system, and perceived usefulness are linked to **satisfaction with and use of the system**.
Shahreki et al. (2020).	167 HR personnel	Malaysia	Clarity of HRIS objectives User satisfaction User support UTAUT variables (Venkatesh et al., 2003): Social influence and facilitating conditions	Perceived ease of use, perceived usefulness, clarity of HRIS objectives, user satisfaction, support with use, social influence, and facilitating conditions are directly and positively correlated with **intention to use the system**.

1 *According to the authors, although the system is not perceived as useful, because its use is obligatory, they use it*.

**Table 2:** Synthesis of studies based on the UTAUT model (Venkatesh et al., 2003) to explain the acceptance of a technology by users.

**Source**	**Sample**	**Country**	**Proposed extension of the UTAUT model**	**Main results**
Heikkil and Smale (2011)	18 HR managers	Europe	Linguistic normalisation	Linguistic skills in English predict **effort expectancy and use of the system (standardised in English)**. Linguistic competence and standardisation of the tool in one international language positively predicts **performance expectancy**. Social influence and facilitating conditions may reduce the role played by **linguistic standardisation in the intention to use the system**.
Yoo et al. (2012)	226 employees in the catering industry	South Korea	Intrinsic and extrinsic motivation	Perceived ease of use, perceived usefulness, social influence and absence of anxiety encourage **a positive attitude to the HRIS**. Intrinsic motivation (effort expectancy, attitudes, and absence of anxiety) predicts **intention to use**. Extrinsic motivation (perceived ease of use, facilitating conditions, and social influence) predicts **intrinsic motivation**.
Lassoued and Hofaidhllaoui (2013)	392 employees of the postal service	Tunisia	Self-image	Effort expectancy, self-image in the eyes of others, facilitating conditions, and involvement of the management are positively correlated with **intention to use**. On the other hand, performance expectancy, social influence, and perceived control do not significantly predict **intention to use**.
Harindran and Jawahar (2016)	40 public sector managers	India	General positive or negative affect.	Affective state (positive or negative) predicts **performance expectancy and effort expectancy**. Performance, effort expectancy and affective state are predictors of **intention to use**.
Rahman et al. (2016)	300 employees from the banking and financial sector	Bangladesh	No extension of the UTAUT model.	Social influence is predictive of **intention to use the HRIS**. Intention to use is a partial mediator of the relationship between social influence and **planned use**. Intention predicts the probability of the **planned use**. **Intention and planned use** are not significantly predicted by facilitating conditions, performance expectancy, or effort expectancy.
Noutsa et al. (2017)	268 HR personnel	Cameroon	Ulrich's model of Human Resource roles (Ulrich, 1997) Information systems success model (Delone and McLean, 1992) Determinants of user satisfaction according to (Haines and Petit, 1997)	Perceived quality of the system predicts **effort expectancy**. Effort expectancy predicts **performance expectancy**. Effort expectancy and perceived quality of the system predicts **satisfaction**. **Satisfaction** and **use** are strongly interrelated. The variables of the UTAUT model do not enable prediction of the actual use of the tool. But the quality of the system and satisfaction predict **the use of the tool**. Voluntary use is a condition for **social influence predicting different measures of acceptance and use of the tool**.
Mahfod et al. (2017)	87 HR personnel	Bahrain/Jordan	No extension of the UTAUT model.	Facilitating conditions predict **effort expectancy**. Social influence predicts **performance expectancy**. There is no predictive effect of the variables of the UTAUT model on **attitude toward the system**.

### Synthesis of the Literature on the Acceptance of HRIS Having the TAM as a Theoretical Basis

**Figure 1:**
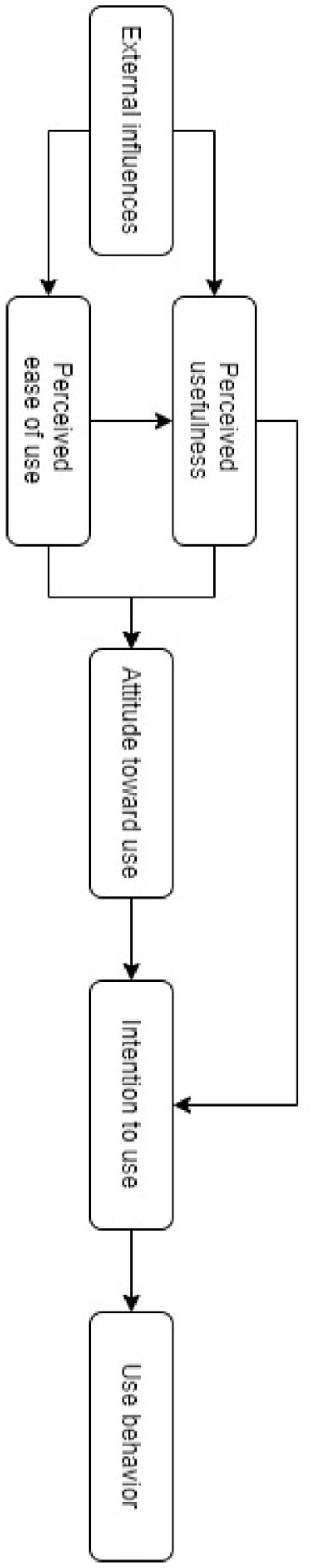
Technology acceptance model (TAM)—(Davis, 1989).

The technology acceptance model (TAM) was proposed by Davis in 1989. This model is based on the theory of reasoned action by Fishbein and Ajzen (1975). For Davis, the a priori evaluation of perceived usefulness and perceived ease of use influences the attitude toward use of the technology in question. This attitude predicts the intention to use the system. In its turn, intention is the trigger for actual use behaviour (Figure 1). The user will perceive the system as useful if they think that it can help them to improve their performance in their job. The same applies if they perceive it as easy to use, that is to say it requires little effort to master (Davis, 1989). The overall ecosystem, external to use, can intervene in that it can facilitate these two explanatory variables (due to the initial training of employees, and economic and strategic aspects for the organisation).

In the previous table, we present the main results present in the articles based on the TAM in our corpus, taking care to emphasise the effects of the variables already incorporated in the TAM, and the effects of the variables added to it in the different studies listed. To facilitate reading of the previous synthesis, outcomes are indicated in bold.

To summarise this table, it is noticeable that the results in the literature on the acceptance of Human Resource Information Systems tend to confirm the predictive power of perceived usefulness and perceived ease of use (Huang and Martin-Taylor, 2013; Saleh and Saleh, 2016; Kamaludin and Kamaludin, 2017; Bayraktaroglu et al., 2019). This observation is consistent with the conclusions obtained by (King and He, 2006). These authors demonstrated that the variables of perceived usefulness and perceived ease of use produce consistent effects in various situations of use and with different types of users. Our observation is in keeping with the robustness of the TAM model.

We can also note that certain more contextual variables seem relevant for the study of the acceptance of HRIS. Indeed, as Amiel and Van De Leemput (2014) show, the national context also influences the results. They demonstrated that, for the same HRIS system, users in the USA perceived it as less easy to use and less useful, yet they used it more frequently than European users. Other researchers, who chose to take into account the valuing of respect and obedience in the national culture, added to the model the social influence exercised by the other employees and obtained significant results (Kamaludin and Kamaludin, 2017). These observations agree with those of Nistor et al. (2013) having identified within the framework of the learning system Hofstede's dimensions of national culture as variables predictive of effort and performance beliefs, behavioural intention and actual use.

Several authors have also proposed incorporating Ulrich Model of Human Resource roles (1997) into the TAM model (Voermans and van Veldhoven, 2007; Panos and Bellou, 2016). In their study, Voermans and Van Veldhoven in 2007, showed that if employees and managers had a preference for the role of strategic partner or administrative expert, then their attitude toward the HRIS would tend to be more positive, while if the preference was for the employee support role, the attitude would tend to be more negative. Panos and Bellou, in 2016, focused on the results obtained by the HRIS. They showed a relationship between the goals of the HR department and the results achieved by the latter moderated by the attitude to the system.

### Synthesis of the Literature on the Acceptance of HRIS Having the UTAUT Model as a Theoretical Basis

**Figure 2:**
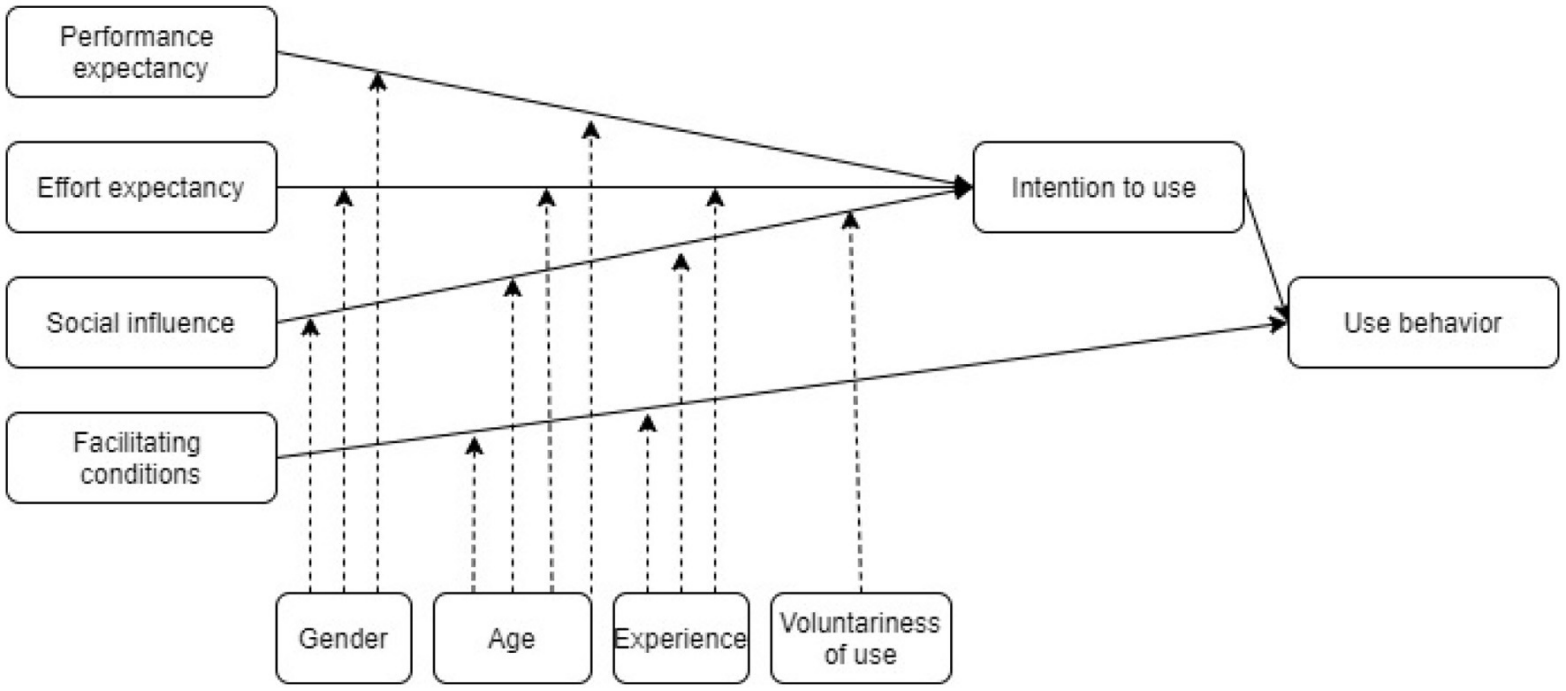
Unified theory of acceptance and use of technology (Venkatesh et al., 2003, p. 447).

The unified theory of acceptance and use of technology (UTAUT) was constructed on the basis of the combination of several models including the TAM (Venkatesh et al., 2003). This theoretical model incorporates social influence and facilitating conditions as explanatory variables, in addition to effort expectancy (perceived ease of use) and performance expectancy (perceived usefulness). Social influence is made up of two different aspects: support from management and the organisation on the one hand; belief on the part of the user that the team members who are important to them would value the use of the system on the other hand. Facilitating conditions include the resources and the knowledge necessary for using the system, the compatibility of this system with the others already used, and the availability of a person or a group to assist the user when needed (Venkatesh et al., 2003). The integration of these two variables takes UTAUT models beyond the framework of the theory of reasoned action and moves it closer to the theory of planned behaviour proposed by Ajzen (1991). Finally, the authors include in their model the moderating variables of gender, age, experience with technology and voluntary use (Figure 2). We should note here that in the context of HRIS, voluntary use has rarely been studied, because technology is most often deployed by the employer and is therefore rarely a matter of individual choice, unlike other technologies.

As with the articles in our corpus dealing with the TAM model, we propose in the previous table a synthesis of the main results of articles dealing with the UTAUT model. This table presents the extensions proposed by the authors, as well as the effects of the variables of the UTAUT models and the extensions they propose.

The results published in the theoretical context of the UTAUT model are more qualified (Table 2) than those published for the TAM model (Table 1). Indeed, some studies did not reveal any significant predictive link between perceived effort or performance expectancy, with the intention to use (Rahman et al., 2016; Mahfod et al., 2017; Noutsa et al., 2017). On the other hand, they underlined the importance of other predictors, such as quality of the system, for example (Noutsa et al., 2017). Other studies partially confirm the predictors arising from the earlier TAM model, such as effort expectancy, but not performance expectancy (Lassoued and Hofaidhllaoui, 2013). These inconsistent results can be attributed to interactions between the explanatory variables. For example, Mahfod et al. (2017) showed that, when facilitating conditions were perceived as poor, this had an impact on the perception of effort expectancy. Likewise, they demonstrated that social influence predicted performance expectancy. This nuance in the results leads us to consider the importance of expanding the UTAUT model. Indeed, it is foreseeable that these differences in terms of results in the context of HRIS can be explained by the role played by other variables which are not included in the UTAUT. For example, Noutsa et al. (2017), showed that effort expectancy was a predictor of satisfaction and that satisfaction with an HRIS in turn predicted use of the technology. Therefore, effort expectancy does not appear to be a direct predictor of use but rather a variable related to one of its most direct predictors, namely, satisfaction. On the other hand, results concerning the quality of the system lead us to consider the integration of characteristics of the tool as potential explanatory variables of the acceptance of HRIS. For these authors, satisfaction with the use of HRIS functionalities is a key element in its actual use and in achieving the strategic objectives of HR departments.

### Synthesis of the Principal Variables of Interest for Studying the Acceptance of Human Resource Information Systems

After presenting the results obtained via the TAM and UTAUT models, in the context of the study of the acceptance of HRIS, we propose to analyse the variables which have been added to these models in this specific context. We will select those presenting congruent results in at least two studies. This choice permits to retain reliable elements and to discuss at least five elements. Three of which are at the level of technological characteristics, one at the individual level and one at the organisational level.

As demonstrated by Tables 1, 2, in the context of HRIS, the TAM and UTAUT models have been combined with others including, for example, the Yale model of communication and persuasion (Saleh and Saleh, 2016); the Ulrich model of HR department roles (Voermans and van Veldhoven, 2007; Panos and Bellou, 2016; Noutsa et al., 2017): the Information Systems success model of Delone and MacLean (Kolatshi, 2017; Noutsa et al., 2017; Bayraktaroglu et al., 2019) and the Haines and Petit explanatory model of HRIS success (Bamel et al., 2014; Noutsa et al., 2017). On the other hand, some authors have added explanatory variables to these two models, such as motivation (Yoo et al., 2012), emotional state (Harindran and Jawahar, 2016), the user's country (Amiel and Van De Leemput, 2014) and the impact of linguistic normalisation (Heikkil and Smale, 2011).

With regard to these different extensions of the models, many factors can therefore be included to explain the acceptance of a technology dedicated to the management of human resources. The acceptance of an HRIS is of course partly explained by its perceived usefulness (i.e., performance expectancy) and its perceived ease of use (i.e., effort expectancy), as these two variables are incorporated in the “basic” models. But, in view of the literature review which we have just proposed, it seems important to consider other factors in order to explain, even, the intention of use. We propose to group and present these factors on the basis of 3 categories: technological, individual and organisational.

First of all we propose to tackle the variables relative to the specific characteristics of the technology. In fact, the quality and wealth of the data contained in the HRIS influences its perceived usefulness, perceived ease of use and intention of use (Huang and Martin-Taylor, 2013; Kamaludin and Kamaludin, 2017; Kolatshi, 2017; Udekwe et al., 2017). Among the characteristics of the technology, the overall quality of the computer system (Noutsa et al., 2017; Bayraktaroglu et al., 2019), its security (Saleh and Saleh, 2016; Udekwe et al., 2017) and its processing speed (Huang and Martin-Taylor, 2013; Bamel et al., 2014; Saleh and Saleh, 2016; ) are linked to intention of use and its usual predictors. Consequently, we propose to consider most particularly in the analysis of the acceptance of HRIS the quality of information contained in the system, its security and speed of response, as these dimensions present congruent results in at least two studies.

As technology is accepted by individuals, the authors have also proposed individual variables to explain the acceptance of an HRIS, including satisfaction with the system. One of the hypotheses supported by the TAM and UTAUT models is that satisfaction with existing technology is a mediator between psychosocial factors and use (Kolatshi, 2017; Bayraktaroglu et al., 2019). The results for the mediating role of satisfaction strongly suggest a circular causality: the more satisfied a user is with the technology, the more they tend to use it and the more they use it the more they are satisfied with it (Kamaludin and Kamaludin, 2017; Noutsa et al., 2017). Satisfaction with an HRIS appears to be particularly important in this fully computerised profession: if a Human Resource professional is satisfied with the HRIS they have to use, they will also have less intention of leaving the company (Kolatshi, 2017). On the other hand, skill and experience with the technology has a predictive power which varies from one study to another. In fact, studies have demonstrated that someone skilled in the use of the system and with previous experience in technology will be more likely to accept an HRIS (Panayotopoulou et al., 2010; Bamel et al., 2014; Alam et al., 2016; Udekwe et al., 2017) while other authors did not observe any significant link (Voermans and van Veldhoven, 2007; Heikkil and Smale, 2011; Kolatshi, 2017). It is very probable that earlier experience only plays a limited role when the introduction and use of HRIS is imposed by the company on its users and they are left no choice other than to use it. We have identified via different articles individual variables which can affect the intention to use HRIS: the affective state of the user (Harindran and Jawahar, 2016); the need for validation (Lassoued and Hofaidhllaoui, 2013); confidence in technology in general (Bamel et al., 2014) and finally command of the language used in the system (Heikkil and Smale, 2011; Huang and Martin-Taylor, 2013). These variables can lead to the perception of HRIS as more complex to use and as less useful. While it is argued that these variables affect the intention to use, there is nevertheless no evidence that they affect the frequency of actual use (Amiel and Van De Leemput, 2014). Thus, apart from users' satisfaction with the HRIS, the results of which are congruent in the various studies that have considered it, we will not retain the aforementioned individual variables.

It seems important to qualify the integration of these individual variables: they have often only been tested sporadically and in a restricted national and cultural context, whereas the acceptance of a technology is very likely dependent on the economic, legal, cultural, and linguistic context (Strohmeier and Rudiger, 2009; Panayotopoulou et al., 2010; Amiel and Van De Leemput, 2014). Indeed many authors agree in saying that individual acceptance can be affected by the country in which the new technology is implemented (Ramirez, 2002; Strohmeier and Rudiger, 2009; Alam et al., 2016; Kamaludin and Kamaludin, 2017; Bayraktaroglu et al., 2019). For example, social influence has a substantial impact in Asian countries (Rahman et al., 2016; Kamaludin and Kamaludin, 2017) and in the Arab world (Lassoued and Hofaidhllaoui, 2013; Kolatshi, 2017; Mahfod et al., 2017) while this relationship is disputed in many studies carried out in Western countries which are in all probability more individualist (Kamaludin and Kamaludin, 2017).

Finally, the effects of gender, age and education as moderators of acceptance, although present in the UTAUT model, are variable, with a tendency not to present any significant influence (Voermans and van Veldhoven, 2007; Panayotopoulou et al., 2010; Abdulah et al., 2013; Kolatshi, 2017). The rejection of hypotheses linked to gender, age and education can be explained by the fact that these hypotheses have “aged” and are thought nowadays to be less decisive according to Strohmeier and Rudiger (2009). This hypothesis is however controversial as these moderators still show effects in studies subsequent to the proposal of these authors (Venkatesh et al., 2016).

Beyond the characteristics of the technology and individuals, variables relative to the organisational context have also been drawn on in earlier studies, which we propose to incorporate more systematically into the analytical framework, in an approach based on social psychology, and the psychology of work and organisations. First of all, with regard to the organisation, several authors have supported the idea that support from the company's management encourages use and intention to use (Lassoued and Hofaidhllaoui, 2013; Alam et al., 2016; Kolatshi, 2017; Udekwe et al., 2017). We should note that management support is one component of social influence of the UTAUT model, the second being peer influence. Studies that added management support as a variable did not include peer influence. We cannot therefore determine whether the management support component should be isolated in future studies or whether it is indeed a single common mechanism of social influence. Appropriate organisational communication seems to promote the acceptance of HRIS (Huang and Martin-Taylor, 2013). The role of the Human Resources Department and the expectations of employees toward it also seem to be variables which influence the acceptance of HRIS (Voermans and van Veldhoven, 2007; Panos and Bellou, 2016). Voermans and van Veldhoven (2007) propose the inclusion of expectations of human resource functions as a determinant of the use of technology. So, based on Ulrich model (1997), they demonstrated that when the HR roles expected by employees within the organisation are those of strategic partner or administrative expert, the attitude toward the HRIS is more positive. Conversely, when expectations revolve around a role focused on support for employees and monitoring quality-of-life at work, employees such as HR Managers have a more negative attitude toward HRIS. Support from management and the role of the HR Department seem to help explain variations in the acceptance of HRIS, and in whether or not the results are achieved. This point is important because it represents both a specificity of HRIS compared to other technologies, and a key organisational dimension.

In the same vein, it is observable that in the context of HRIS, compared to other technologies, the results concerning the effect of variables explaining acceptance, are less consistent. Indeed, as we stated previously, the performance expectancy and effort expectancy variables present the expected effects less consistently in our corpus, unlike what can be observed in other literature reviews. not directly related to Human Resource Information Systems (Khechine et al., 2016; Venkatesh et al., 2016).

Following this review, we support the necessity in forthcoming studies on the acceptance of HRIS to link the technological, individual and organisational levels in the analysis, while taking into account the specificities of HRIS. It is this point of view that we will develop and defend in the following discussion.

## Discussion and Perspectives

### Variables Identified as Relevant for Studying HRIS Acceptance

HRIS are professional software programs which play a strategic role within organisations. The traditional models such as UTAUT and TAM which account for motivations for using technologies, although relevant, could be extended to help us to understand all the determining factors for these systems. The aforementioned studies have proposed numerous extensions to these traditional models independently of each other and although some of these variables demonstrated an explanatory power, they have not been summarised in a deliberation dedicated to Human Resource Information Systems.

We will first summarise the elements that we believe are scientifically relevant, at 3 levels: technological, individual, and organisational.

In terms of technology, existing research suggests taking into account its perceived characteristics: quality of information, security and speed in meeting need, which appear to be decisive in the acceptance of HRIS.

At individual level, satisfaction with HRIS appears to be one of the most directly predictive factors of their acceptance and continued use.

Finally, at organisational level, we propose to take account of two categories of variables. A first category is linked to national and cultural characteristics. Indeed, as we have already stated, the results obtained differ from one country to the next. In particular it appears that in Asian countries, normative pressure (colleagues and senior colleagues) plays a more important role in acceptance by employees. Likewise, Strohmeier and Rudiger (2009) have demonstrated that the adoption of software dedicated to the management of human resources differs in the countries of Western Europe and the countries of Eastern Europe, where they are more often used. We only found a few studies that enabled a comparison of the acceptance of HRIS on the basis of the countries in which they are deployed, but we can assume that such comparisons are relevant given that they have already furthered the subject of technology acceptance in the educational field. Forthcoming studies could therefore foresee the inclusion of international or intercultural comparisons which would allow these factors to be tested. Such research work would enable us to understand the variations in the acceptance of HRIS linked to sociocultural aspects (collectivism-individualism, uncertainty control, hierarchy gap, Hofstede, 2011), legislative aspects (the complexity of labour law and legislation on data protection) and economic aspects (level of development of the country, its efforts in investment and innovation).

A second category of organisational variables relates to the question of the expectations of the role of HR departments, which also seem worth taking into account in terms of the consistency of the results obtained (Voermans and van Veldhoven, 2007; Panos and Bellou, 2016; Noutsa et al., 2017).

### Perspective of the Results Through the Model of the Human-Machine-Organisation Symbiosis

To encompass these 3 levels (technological, individual, and organisational), the Human-Technology-Organisation Symbiosis model appears appropriate, as it is able to account for a pre-existing relationship between the employee and the information system in the field of HRIS (Brangier and Hammes-Adelé, 2011). This model addresses the relationship between Human and technology as a symbiotic relationship in which technology supports and changes the way humans operate. In return, humans adapt and develop technology in order to respond to the new habits and needs generated by the use of the latter. So, technology transforms Human and their environments, leading them to change their needs and therefore to transform technology as a result so that it adapts to the new needs which it has partly generated. This reactive cycle leads to a dependence of Human on technology and of technology on Human. HRIS are designed to assist managers in human resource departments in their routine tasks and decision-making. Therefore, insofar as certain tasks are delegated or facilitated by HRIS, managers may take on new roles such as strategic partners and therefore develop new needs for technological assistance. These new needs then lead to the design of new functionalities in the HRIS requiring the implementation of additional data. The repetition of this loop as HRIS technologies are developed and used could therefore be seen as a factor in user satisfaction with the system. So a really mutual collaboration develops between the HRIS and human resource managers. By facilitating access to information, by assisting management and by reducing the time devoted to administrative tasks, HRIS allow managers to focus on the strategic and human aspects of their profession. As an example, a human resource department which has established a paperless absence management system, can then devote more time to improving analysis of the absence rate and therefore needs its IT tool to be capable of providing more detailed analyses of these rates.

This symbiotic relationship also implies the organisation (Brangier et al., 2010). Indeed, professional needs mainly ensue from tasks prescribed and induced by the organisation, and these paperless processes are most often defined by the organisation's strategic decision-makers depending on the organisation and its culture. The organisation and its strategy, notably in terms of the role of HR departments, are modelled and standardised during the design of an IT solution for Human Resource Management, and the information systems supporting it. It seems likely that HRIS allow organisations to better define, manage and pursue an HR policy in the long-term, or even to free up time in order to extend and improve the quality of their HR services to employees. Therefore, as revealed by the symbiosis model, there is a reaction of the three components to each other. The systems are designed to meet the needs of human resource departments, themselves induced by the organisation. When the system is deployed in the organisation, it transforms its way of functioning. It can both freeze the organisation's ways of operation and enable the emergence of new tasks for professionals. These new tasks in their turn generate new requirements in terms of functionality and lead to the development and evolution of HRIS. Thus, the use of the HRIS leads to the evolution of the role of the organisation's human resources department. In order to maintain user satisfaction with the system and therefore its use, it becomes necessary to develop it by taking into account the new expectations generated by this evolution of roles. In order to be usable, these new functionalities will require the implementation of additional data in the system, the quality of which seems to be a predictive factor of the use of the technology. Similarly, the implementation of this new data will raise questions about the consistency between the level of confidentiality of these data and security of the system, as well as the ability of the HRIS to maintain a reasonable response time despite the changing volume of processing it performs. In view of these elements it would appear appropriate to apply the symbiotic human-technology-organisation model to HRIS, in order to better understand the inter-relationship between these three components of the symbiotic relationship and the factors which make it possible.

A final critical question is what makes the HRIS acceptable socially, in particular amongst employees or their representatives. It is possible that beyond the aspects linked to characteristics of use, the issue may be one of satisfaction with the decisions offered by the HRIS. It is conceivable that what can lead employees or their representatives to accept HRIS may be a feeling of procedural and informational justice vis-à-vis decisions taken (Zhou, 2016). As such, HRIS could be seen as offering more reliable, fair and impartial decisions than those of a manager or HR manager (Leo and Huh, 2020).

### Perspective for Future Research

This synthesis has allowed us to confirm the robustness of the general models and the value of considering the variables relating to the quality of the technology as well as the interaction between satisfaction and actual use for understanding the acceptance of HRIS. The value of these variables has been demonstrated for other technologies, so it does not seem to be specific to HRIS (Venkatesh et al., 2016). On the other hand, the impact of the expected role of HR departments on acceptance and the achievement of objectives via technology is an element specific to HRIS technologies. This specificity can be explained in view of the purposes of the technology. Indeed, here it is not the technical characteristics of the machine or the individual perceptions of the machine that will predict its acceptance, but the relationship between the user and the organisational context of use of the machine. Based on this observation, we can hypothesise that the operationalisation of external influences could be the subject of theorisation specific to the objectives pursued by the technology. Future research on the acceptance of Human Resource Information Systems could therefore incorporate contextual dimensions adapted to the HR field such as organisational justice This would then help us to broaden our understanding of the acceptance of Human Resource Information Systems and go beyond the study of the link between individual and technological levels, already thoroughly expanded on in the TAM and UTAUT models. By including the organisational level in the analysis, currently little taken into account, we could go beyond the vision of an self-determined subject, deciding, and acting outside of social and organisational restrictions, which seems to emerge from the theory of reasoned action of Fishbein and Ajzen, which is the foundation of the TAM and UTAUT models. While the TAM model does incorporate the idea that there are “external influences,” it does not operationalise them and therefore does not really incorporate them into the functioning of the model. Following Brangier (2003), who considers it necessary to understand the interdependencies between the “technological, organisational and cognitive dimensions,” and based on an approach rooted in the social psychology of work and organisations, we argue that the organisational level must be linked to the individual and technological levels in the analysis of acceptance. We believe that the structure of the organisation, like its culture, are organisational elements of interest that can have an impact on actual use. We also argue that it is important to consider a feedback loop between actual use and organisational context, which can be modified by these actions and, which can, in turn, influence or partially predict actual use.

### Contribution to the Field and to Decision-making

With regard to social relevance, as the use and therefore the acceptance of the system is one of the levers for the development of technologies and their commercialisation, this wider understanding would be beneficial to publishers as well as to users. Indeed, understanding the various factors that make HRIS acceptable could allow us to design solutions which are more respectful of the expectations and requirements of their users. We will develop our argument in the same vein as previously, making suggestions at technological, then individual and finally organisational levels.

In terms of technical characteristics, our study highlighted the importance of the responsiveness of the system (Voermans and van Veldhoven, 2007; Bamel et al., 2014; Saleh and Saleh, 2016). It therefore appears, for example, that particular attention could be paid to scalability, which allows the system to adapt to changes in the number of requests and thus maintain a rapid and stable response time. The same applies to aspects of security (Voermans and van Veldhoven, 2007; Saleh and Saleh, 2016), the importance of which in acceptance by users could be boosted by legal developments, with in particular the General Data Protection Regulation in Europe and the Privacy Shield in the United States. The quality of the system also includes the quality of the information implemented within it. This data quality appears to be an element which encourages the use of HRIS (Huang and Martin-Taylor, 2013; Kamaludin and Kamaludin, 2017; Kolatshi, 2017; Bayraktaroglu et al., 2019). It therefore appears that special attention should be paid by the company or its HR department to the quality of the data implemented in the software.

In terms of the individual, and with regard to the importance of satisfaction with the tool in user behaviour (Kamaludin and Kamaludin, 2017; Kolatshi, 2017; Noutsa et al., 2017; Bayraktaroglu et al., 2019), it appears to be important that companies examine this satisfaction some time after the deployment of the system. This measure is relatively simple to operationalise and seems to provide reliable information on the continued use of the system (Bayraktaroglu et al., 2019). Companies could then anticipate potential failures of these deployments and so take measures to correct the dissatisfaction of the various employees.

Finally, as regards factors dependent on the organisation, it appears that the communication and the objectives set for the use of the HRIS must be defined at company level and include the entire management of the company rather than emanate solely from the HR department. On the one hand, the support of management as a whole should not be neglected in view of the consistency of its effect on the acceptance of technology (Lassoued and Hofaidhllaoui, 2013; Bamel et al., 2014; Alam et al., 2016; Kolatshi, 2017). On the other hand, as the expectations of employees toward the role of their HR department have proven effects on the acceptance of HRIS (Voermans and van Veldhoven, 2007; Panos and Bellou, 2016), it seems relevant to consider these expectations when designing the services offered by these professionals. For example, when employees expect their HR department to play a supportive role, they have a more negative attitude toward an HRIS with a purely administrative focus (Voermans and van Veldhoven, 2007). In this case, one of the criteria for choosing the system could be its ability to simplify interactions between the HR department and employees so that it boosts the response provided by HR departments to employees' requests. The deployment of an HRIS sometimes involves the intervention of a choice assistance company or support for employees with the digital transition. This help and support can be an opportunity to consult the expectations of stakeholders (decision-makers, HR managers, but also employees who will be required to enter information into the system). The expected role of the HR department could also serve as a guide for defining the schedule for deploying HRIS functionalities.

As satisfaction is an important element of use, the HR role of monitoring employees' quality of life at work could be the subject of specific functionalities which would make HRIS more attractive. Another argument in favour of HRIS is that it can lead to more transparent, fair, and equitable decisions for employees.

### Limits and Perspectives

The main limitation of this literature review is that it is not systematic. Although it identifies factors relevant to the study of HRIS acceptance, it is conceivable that studies not based on the TAM and UTAUT models could further enrich these propositions. Indeed, the perspective adopted in this review is only one possible angle among others and future research would benefit from completing the different scales of analysis in order to understand acceptance in its complexity. In particular, it could include the views of computer and management sciences, thus providing a more comprehensive view of the issue of HRIS acceptance, its antecedents and its outcomes (Iqbal et al., 2019).

The definition of acceptance and the predictive role of the intention to use on effective use have already been the subject of much criticism. Our review is based on acceptance in the sense of the TAM and UTAUT models, so the same limitation applies. Indeed, the predictive role of intention on usage has only been studied to a very limited extent by the articles presented in the corpus of this article. Some of the studies focus on the use of existing technologies, others on the intention to use technologies under development, and very few studies have correlated intention to use with effective use in a longitudinal research. Acceptance is in fact only one stage in a more complex cycle, from conception of the need by the organisation to an effective and sustainable use. This cycle goes through various stages (perceptions, intentions, acceptance, and effective use) and needs feedbacks in response to changing needs or external events (mergers, acquisitions, reorganisation of the company, etc.). To our knowledge, the global understanding of the interaction cycle between the organisation, the system and the users is rarely studied in the HRIS literature, which has often focused on a single stage of this cycle. As HRIS has now become a key system in the IT landscape of companies, we believe it is important that future research should examine the entire HRIS life cycle through longitudinal or retrospective research. These researches could be conducted with human resources managers, or IT providers, to reveal the adjustments made to these systems, to adapt to and satisfy the people and the needs of the organisation.

## Author Contributions

LM: constitution of the corpus of the article, preliminary drafting, and revision. DG: critical review involving a significant contribution to intellectual content. CS: critical review involving a significant contribution to intellectual content. All authors contributed to the article and approved the submitted version.

## Conflict of Interest

The authors declare that the research was conducted in the absence of any commercial or financial relationships that could be construed as a potential conflict of interest.

## Footnotes

^1^As an illustration of this representativeness, Google Scholar lists just over 55,000 citations for the article in which Davis proposes the TAM (Davis, 1989). For the article that made it possible to propose the UTAUT (Venkatesh et al., 2003) the number of citations listed stands at almost 31,000.
